# Consequences of the variability of the CovRS and RopB regulators among *Streptococcus pyogenes* causing human infections

**DOI:** 10.1038/srep12057

**Published:** 2015-07-15

**Authors:** Ana Friães, Catarina Pato, José Melo-Cristino, Mario Ramirez

**Affiliations:** 1 Instituto de Microbiologia, Instituto de Medicina Molecular, Faculdade de Medicina, Universidade de Lisboa, Lisbon, Portugal

## Abstract

To evaluate the importance of *covRS* and *ropB* mutations in invasive disease caused by Group A Streptococci (GAS), we determined the sequence of the *covRS* and *ropB* genes of 191 isolates from invasive infections and pharyngitis, comprising a diverse set of *emm* types and multilocus sequence types. The production of SpeB and the activity of NAD glycohydrolase (NADase) and streptolysin S (SLS) were evaluated. The results support the acquisition of null *covS* alleles (predicted to eliminate protein function), resulting in downregulation of SpeB and upregulation of NADase and SLS, as a mechanism possibly contributing to higher invasiveness. Among the isolates tested, this mechanism was found to be uncommon (10% of invasive isolates) and was not more prevalent among clones with enhanced invasiveness (including M1T1) but occurred in diverse genetic backgrounds. In lineages such as *emm*64, these changes did not result in upregulation of NADase and SLS, highlighting the diversity of regulatory pathways in GAS. Despite abrogating SpeB production, null alleles in *ropB* were not associated with invasive infection. The *covRS* and *ropB* genes are under stabilising selection and no expansion of isolates carrying null alleles has been observed, suggesting that the presence of these regulators is important for overall fitness.

*Streptococcus pyogenes* (Group A Streptococci, GAS) is a human pathogen that can asymptomatically colonize the oropharynx, but is also responsible for a variety of human diseases, ranging from uncomplicated superficial infections of the respiratory tract and skin, such as pharyngitis and impetigo, to severe invasive infections associated with high morbidity and mortality, like necrotizing fasciitis and streptococcal toxic shock syndrome (STSS)^1^.

The recognition of the ability of strains belonging to the highly invasive M1T1 clone to acquire mutations in the *covRS* genes during skin and soft tissue infection in mice suggested that GAS could increase its capacity to invade deeper tissues by altering its regulatory networks in order to produce a switch to an invasive transcriptome profile^2^,^3^. The adjacent and cotranscribed genes *covR* and *covS* encode the two-component regulatory system CovRS (also known as CsrRS). The DNA-binding response regulator CovR acts mostly as a repressor of transcription upon phosphorylation by the sensor kinase/phosphatase CovS, which responds to stress factors, such as elevated temperatures, high saline concentrations, and decreased pH^4^. CovRS is estimated to directly or indirectly influence the expression of 10–15% of the GAS genome. Although it is also involved in the regulation of metabolic genes, it is mostly known for controlling the expression of a large number of genes encoding factors that promote GAS virulence and evasion of the host immune system^5^,^6^. Most studies indicate that mutations impairing CovRS function result in the upregulation of genes encoding the hyaluronic acid capsule, streptolysins S and O, streptokinase, DNases, the interleukin-protease SpyCEP, and NAD-glycohydrolase (NADase), among others, and in the downregulation of proteins like the extracellular cysteine protease SpeB and the protein-G-related α2-macroglobulin-binding protein GRAB^7^,^8^. However, contrasting results have been reported regarding the effect of *covRS* mutations in some of those virulence factors, which may be partly explained by the interaction of CovRS with other transcriptional regulators, resulting in complex regulation patterns that can vary between different strains^8^,^9^.

Mutations in CovRS and in the stand-alone transcriptional regulator RopB have been identified in isolates recovered from human infections^7^,^10^,^11^,^12^. RopB (also known as Rgg) is encoded by the *ropB* (*rgg*) gene, which is located 940 bp away from *speB*, in the opposite DNA strand, and directly binds the promoter of the latter gene to activate its transcription^13^. In some strains, this regulator has been reported to affect the transcription of other virulence factors, including DNases, the hyaluronic acid capsule, NADase, streptokinase, streptolysins, and phage-encoded superantigens, among others^10^,^11^.

The downregulation of SpeB, which is usually observed as a consequence of CovRS and RopB mutations, is considered to be a determining step in the transition to an invasive phenotype, since this potent protease degrades several extracellular GAS virulence factors that play an important role in the invasive process, including the M protein, the F1 protein, C5a peptidase, streptokinase, and SmeZ^14^. In agreement, SpeB production has been inversely correlated with disease severity, in both human infections and murine models^15^,^16^. SpeB is initially synthesised and secreted as an inactive 42-kDa zymogen, which is then autocatalytically processed through a series of intermediates to form the 28-kDa mature active protease. Each stage from transcription to mature SpeB involves tight regulatory controls, including post-transcriptional and post-translational mechanisms^14^.

Some studies suggest that *covRS* and *ropB* mutations occur more frequently among isolates from invasive infections, while others found them in a similar proportion among non-invasive isolates^7^,^11^,^12^. Contrasting observations have also been reported regarding the impact of alterations in these regulators on virulence using animal models, as well as on the expression of virulence factors like SpeB and the streptolysins^2^,^3^,^5^,^7^,^8^,^11^,^17^,^18^,^19^,^20^. Given these discrepancies and the fact that most of the studies have focused on particular *emm* types, especially *emm*1 and *emm*3, the importance of the acquisition of these mutations for the overall invasiveness and virulence of GAS strains is still not completely understood.

In order to address these questions, we sequenced the *covRS* and *ropB* genes in a collection of 191 GAS isolates presenting a high diversity of *emm* types and multilocus sequence types (STs), including the most prevalent clones causing pharyngitis and invasive infections in Portugal and in a majority of other countries from temperate climate regions^21^,^22^,^23^. The genetic diversity of the genes was evaluated, and the respective alleles were correlated with the activity of SpeB and of two other virulence factors whose expression has been suggested to be under the influence of CovRS and RopB, at least in some strains, namely the NADase and the streptolysin S (SLS)^7^,^11^,^18^,^24^.

## Results

### Genetic variation of the *covR*, *covS* and *ropB* genes

In the present work, the sequence of the *covR*, *covS*, and *ropB* genes was determined for a collection of 191 isolates (Supplementary Table S1) comprising one third of the isolates of each *emm* type present in a larger collection of strains recovered from pharyngitis and invasive infections in Portugal, which has been characterized elsewhere^21^. In addition, all isolates of *emm* types 1 and 64 were included in this study, since these two *emm* types were shown to be significantly associated with invasive disease.[Table t1]

The three genes presented a high allelic diversity in the studied GAS collection, with Simpson’s index of diversity (SID) values close to the ones obtained for *emm* type and ST (Table 2). The allelic diversity of the *covR* genes was lower than that of *covS* (*P* = 0.0140) and of the ST (*P* = 0.0137). Overall, isolates sharing the same *emm* type or ST frequently shared the same *covR*, *covS*, and *ropB* alleles (Supplementary Table S2). This association was stronger for *covR*, in line with the lower allelic diversity of this gene. These results indicate that the *covR*, *covS*, and *ropB* alleles present in a given isolate were essentially clonal properties, although some level of intra-clonal diversity was observed, particularly for *covS* and *ropB*.

**Figure 1:**
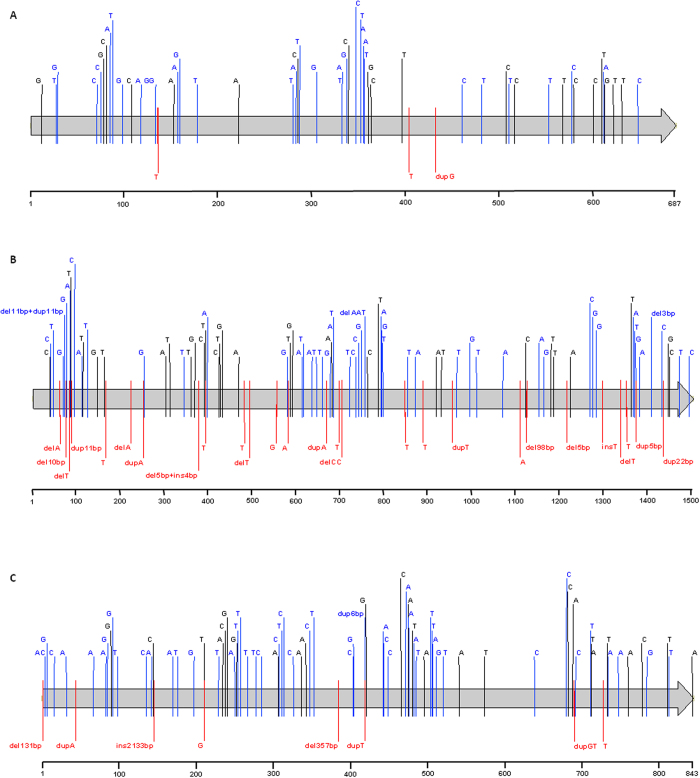
Nucleotide alterations identified in the *covR*(A), *covS* (B), and *ropB* (C) alleles reported in this study and previously deposited in GenBank, relative to the alleles present in strain SF370 (AE004092). Each gene is indicated by a grey arrow and a nucleotide numbering scale is represented below each one. Each sequence variation is indicated in the respective nucleotide position by a letter corresponding to the variant nucleotide, or the sequence or number of base-pairs inserted (ins), deleted (del), or duplicated (dup). Synonymous nucleotide changes are represented in black; missense mutations and short in-frame indels ( ≤6 bp) are represented in blue; changes predicted to result in null alleles, including nonsense mutations, indels that generate frameshifts, and long in-frame indels ( ≥ 357 bp) are represented in red.

Together with the alleles previously deposited in GenBank, we identified a total of 54 alleles for *covR*, 121 for *covS*, and 78 for *ropB* (Table 1). One of the isolates in our study is devoid of the *ropB* gene due to a previously characterized deletion that also involves the *speB* gene^25^. Alterations relative to the nucleotide sequence of the three genes in strain SF370 are depicted in Fig. 1. For each of the genes, more than half of the alleles result in alterations in the amino acid sequence, with *ropB* presenting the highest proportion of distinct amino acid sequences relative to the total number of alleles. However, all the *K*a/*K*s ratios were significantly lower than 1 (Table 1), indicating that all three genes are under stabilising selection. *K*a/*K*s is the ratio of the number of nonsynonymous substitutions per nonsynonymous site to the number of synonymous substitutions per synonymous site. This measure is often used to evaluate deviations from neutral evolution. A value of *K*a/*K*s << 1 indicates that the protein is under stabilising selection, i.e. selection favours alleles that do not change the amino acid sequence. In contrast, a value of *K*a/*K*s >> 1 is strong evidence for positive selection, indicating that selection drove changes to the protein^26^. Nucleotide diversity was also similar for the three genes, with differences in the total number of alleles basically reflecting the different sizes of the genes (687 bp for *covR*, 1503 bp for *covS*, and 843 bp for *rop*B). Null alleles were defined as those presenting changes predicted to result in a complete absence of protein function – including nonsense mutations, indels that result in frameshifts, large in-frame indels ( ≥357 bp), and complete gene deletions. In spite of similar diversity, the *covS* gene presented more null alleles than *covR* (*P* = 0.0004).

### Mutations in *covR*, *covS*, and *ropB*, and association with *emm* type and invasiveness

For *covR* and *ropB*, the reference alleles (those present in SF370) were the most common among the 191 GAS isolates analysed in this study [*n* = 67 (35%), and *n* = 69 (36%), respectively]. The SF370 *covS* allele was present in only five isolates, but 68 isolates (36%) presented only synonymous nucleotide changes (15 alleles). Since our collection was enriched in *emm*1 isolates, the most common *covS* allele (*covS*-02, 28%) included a missense mutation characteristic of the M1T1 clone, namely I332 V. Other missense mutations were also commonly identified in isolates of specific *emm* types, in both *covS* and *ropB* genes.

In addition to the coding sequence of the genes, the region upstream of *covR*, which includes the *covRS* promoter, was also analysed for all isolates, but no nucleotide changes were found in the identified −35 and −10 regions, in the transcription start site, nor in the consensus sequences that have been identified as binding targets for CovR auto-repression^27^.

The overall distribution of *emm* types differed significantly between the isolates carrying missense or null alleles of *covR*, *covS*, or *ropB* and those with no amino acid alterations (*P* < 0.0001). Null alleles were not associated with any particular *emm* type, occurring in isolates of seven different *emm* types for *covS* (SID = 0.909 [CI_95%_ 0.822–0.996]) and of four different *emm* types in the case of *ropB* (SID = 0.867 [CI_95%_ 0.738–0.995]). Missense mutations in *covS* and *ropB* were significantly more prevalent in isolates of certain *emm* types due to the presence of clonal alleles in specific lineages, i.e. alleles carrying amino acid changes that occur in several isolates of that *emm* type (Supplementary Table S1).

According to the SID values, the allele diversity of the three genes was similar for the subsets of isolates associated with pharyngitis and with invasive infections (Table 2). The overall distribution of *covS* and *ropB* alleles was significantly different between the two subsets (*P* < 0.0001), but no specific *covR*, *covS*, or *ropB* allele presented a significant association with either infection type after correcting for the false discovery rate (FDR), except for *ropB*-23, which was significantly associated with invasive infections (*P* = 0.0109). This association is explained by the fact that *ropB*-23 was present in all *emm*64 isolates and not found in any other *emm* type (Supplementary Table S1), and *emm*64 was significantly associated with invasive infections in Portugal^21^. This allele presents only two synonymous nucleotide changes, which are not likely to be the cause of the high invasiveness of this lineage.

The presence of an altered amino acid sequence in general was not associated with infection type for any of the genes. However, null *covS* alleles were significantly overrepresented among the invasive isolates (*P* = 0.0247) (Table 3), even though they were only present in nine of these isolates (10%).

### SpeB production

**Figure 2:**
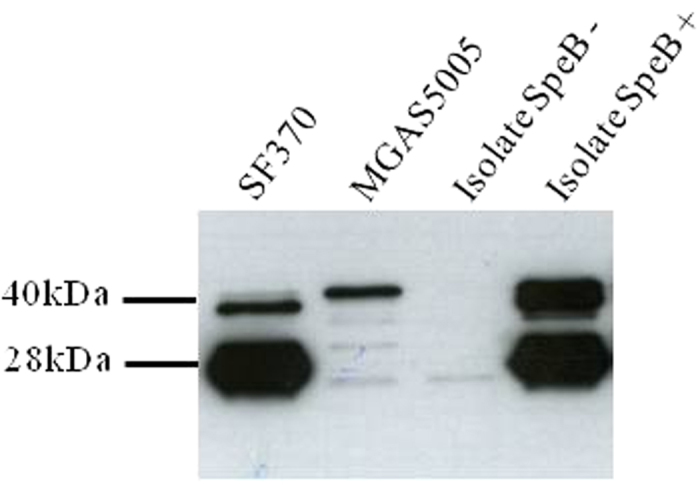
Representative Western blot result for detection of mature SpeB (28 kDa) expression by GAS isolates. The blot shown was cropped to the region of interest.

**Figure 3:**
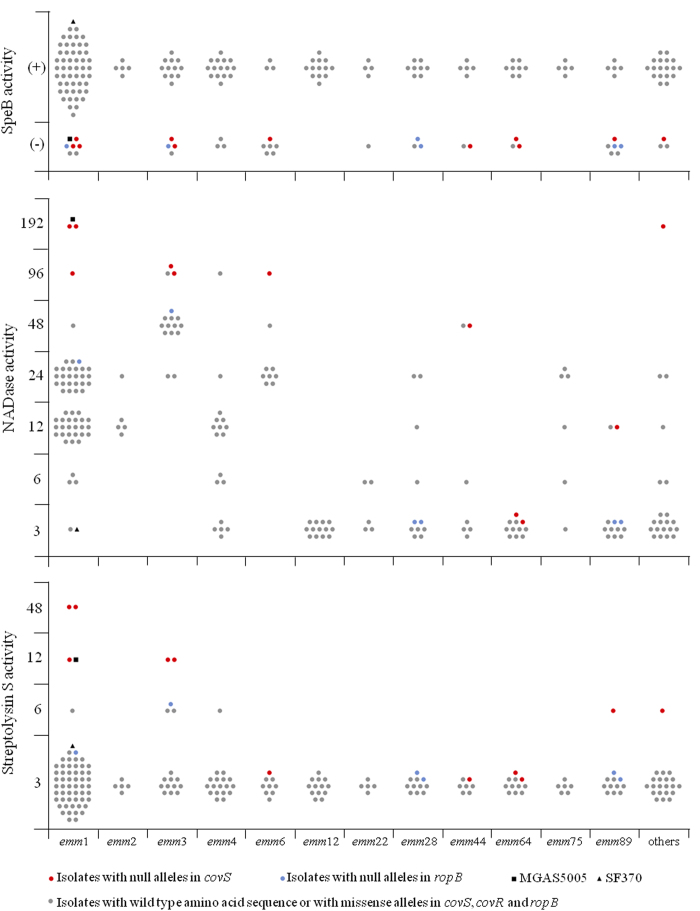
SpeB production and NADase and SLS activity determined for the 191 isolates analysed in this study, according to *emm* type. The *emm* types represented by < 5 isolates are grouped into “Others” and include *emm* 9, 11, 18, 29, 43, 53, 58, 74, 77, 78, 87, 94, 102 and 113. The results of reference strains SF370 and MGAS5005 are also presented.

In this study, the presence or absence of extracellular proteolytic activity was tested for all isolates using a spectrophotometric assay based on the degradation of azocasein by culture supernatants, as well as by a plate assay in which the strains were cultured in solid medium containing casein. To confirm that the proteolytic activity detected by these two methods was essentially due to SpeB, which has been described as the major extracellular protease of GAS^28^, Western blot analysis using monoclonal anti-SpeB antibodies was performed in a subset of six isolates of *emm* types 1, 64 and 89. For each *emm* type, one protease-positive and one protease-negative isolate were randomly chosen. The presence of a 28-kDa band similar to the one detected for SF370 was considered as a positive result (Fig. 2). The Western blot results were concordant with those of the two proteolytic activity assays for this subset of isolates. The *emm*1 strains SF370 and MGAS5005 were used as controls in all assays. As expected, strain SF370, which encodes a functional CovRS, presented proteolytic activity in both assays and was positive for mature SpeB production by Western blotting. In contrast, almost undetectable amounts of mature SpeB and no proteolytic activity were observed for MGAS5005, which harbours a frameshift mutation in *covS* (Fig. 2 and Fig. 3). Taken together, these results confirm that SpeB is the major protease of GAS, allowing us to use the total proteolytic activity as a proxy for SpeB activity. Western blot was also performed for all isolates in which the azocasein and casein-plate results were discordant (*n* = 31). In these cases, the Western blot result was considered as final.

Of a total of 191 isolates, 153 (80%) presented SpeB activity, while for the remaining 38 (20%) no SpeB activity could be detected (Fig. 3 and Supplementary Table S1). All isolates with null alleles, either in *covS* or in *ropB*, were associated with an absence of SpeB activity (*P* < 0.0001 for both). In contrast, the prevalence of missense alleles among the SpeB-negative and SpeB-positive isolates was similar, except for non-clonal missense alleles in *ropB* (i.e. missense alleles that were not identified in other isolates of the same *emm* type in this study or in the GenBank database), which were associated with the absence of SpeB production (*P* = 0.0008). Only two SpeB-negative isolates presented alleles encoding the reference amino acid sequence in all three genes. The absence of SpeB activity in isolates carrying non-null alleles in the three genes could be due to alterations in the *speB* gene, its promoter, or in other mechanisms involved in the post-transcriptional or post-translational regulation of SpeB^14^.

There was no significant association between the SpeB activity of the isolates and infection type. The overall distribution of *emm* types differed significantly between SpeB-producing isolates and those without protease activity (*P* = 0.0030), but after FDR correction, only *emm* types 89 and 6 were significantly associated with the absence of SpeB (*P* = 0.0354 for both).

### NADase activity

In this work, the NADase activity of the isolates was measured by an endpoint titre method based on the degradation of β-NAD by culture supernatants, which results in a reduction of the fluorescence emitted by the reduced form of β-NAD. GAS *emm*1 strains SF370 and MGAS5005 were used as controls of the assay. As expected, SF370 presented a low NADase activity (NADase ≤ 3), while MGAS5005 was found to have the highest NADase activity level measured (NADase = 192) (Fig. 3). The high NADase activity of the latter strain can be attributed to the null *covS* allele, as well as to a different NADase locus from the one carried by old *emm*1 strains such as SF370^29^.

The gene encoding the GAS NADase, known as *spn* or *nga*, presents multiple variants and has been shown to be diverging into NADase-active and -inactive subtypes, which are correlated with *emm* patterns and tissue tropism^30^. Therefore, variations in the levels of NADase activity exhibited by distinct GAS lineages, which may encode different *nga* alleles, are expected and were observed among the isolates analysed in this study (Fig. 3 and Supplementary Table S1). Despite this lineage-specific variation, it was possible to identify a significant association between the highest activity values, namely NADase = 96 and NADase = 192, and the presence of null alleles in *covS* (*P* = 0.0005), while *covR* and *ropB* changes did not significantly contribute to an increased NADase activity. In agreement, all the strains carrying *covS* null alleles expressed higher levels of NADase activity than the majority of the isolates of the same *emm* type, except for two *emm*64 isolates (Fig. 3). The absence of NADase activity could be an intrinsic characteristic of this lineage, regardless of the CovRS alleles, due to alterations in the *nga* gene, its promoter, or other regulatory pathways. It is also possible that *nga* is not part of the CovRS regulon in the *emm*64 clone. Both inter- and intra-serotype differences in the regulatory activity of CovRS have been proposed^9^.

Despite the association between high levels of NADase activity and *covS* null mutations, it was not possible to detect any significant association between the level of NADase activity and invasive disease.

### Streptolysin S activity

The SLS activity of the isolates was evaluated by an endpoint titre method based on the amount of released haemoglobin from sheep erythrocytes during incubation with culture supernatants. Although the majority of the isolates (*n* = 179, 94%) presented an SLS activity ≤ 3, it was possible to identify multiple isolates with increased SLS activity and that also carried alterations in CovRS (Fig. 3). Accordingly, the SLS activity determined for the reference strains SF370 (SLS ≤ 3) and MGAS5005 (SLS = 12) were consistent with a de-repression of the *sag* operon in the latter due to the null allele in *covS*, and a significant association between activity values of SLS = 12 and SLS = 48 and *covS* null alleles was observed (*P* = 0.0003 and 0.0040, respectively). In fact, all isolates with an SLS ≥ 12 presented null alleles in *covS* (Fig. 3 and Supplementary Table S1). Four isolates carrying null alleles and belonging to *emm* types 6, 44, and 64 did not exhibit an increased SLS activity. The coherent low NADase and SLS activity among *emm*64 isolates independent of the CovRS alleles is consistent with a potentially different CovRS regulon in these isolates. Among *emm*6 and *emm*44 isolates, it is possible that the *sag* operon, which encodes SLS activity, is not regulated by CovRS. Alternatively, SLS activity may not be significantly increased in these isolates due to mutations in the *sag* operon, which have been infrequently reported among *S. pyogenes* isolates^31^.

It was not possible to detect any significant associations of SLS activity levels with the presence or absence of changes in *covR* and *ropB*, nor with infection type.

## Discussion

Variations at the level of the regulatory networks governing GAS gene expression may constitute key elements differentiating strains with a high invasive ability from those that carry the same virulence genes, but cause mild infections or asymptomatically colonise the host. Mutations impairing the function of the two-component system CovRS and the stand-alone regulator RopB could play this role and have been shown to be important in animal models of infection, mostly using representatives of the M1T1 clone^2^,^3^,^11^,^17^. However, the actual impact of these mechanisms in invasive human GAS infections remains unclear.

In the characterised collection, all three genes (*covR*, *covS*, and *ropB*) presented a high genetic diversity and the allele distribution was closely associated with *emm* type and ST. Some studies, mostly based on murine infection models, suggest that isolates of the highly invasive M1T1 clone have a higher ability to acquire *covRS* mutations than strains of other *emm* types^17^,^32^. However, in our study mutations in the *covR*, *covS*, and *ropB* genes were not more prevalent in *emm*1 or any other *emm* type. We conclude that among the GAS population causing human infections, the acquisition of mutations in any of the three genes can occur in isolates of diverse lineages, in agreement with data from Japan and Taiwan^11^,^12^. We also did not find a higher prevalence of *covRS* mutations in isolates carrying the DNase gene *sda1* (data not shown), although it has been suggested that the presence of the phage encoding this gene exerts a selective pressure that favours the acquisition of *covRS* mutations, at least in the M1T1 genetic background^33^.

Null *covS* alleles were found to have a significant association with invasive infections, while the missense alleles in *covS* and all null and missense alleles in *covR* and *ropB* were not differently distributed among isolates from pharyngitis and from invasive disease. According to these results, only the null alleles in *covS* would contribute to the transition to invasive infection. In addition, the fact that null alleles are significantly more common in *covS* than in *covR* supports the hypothesis that during the infection process, the acquisition of mutations that impair signalling through CovS, while keeping CovR functional and possibly responsive to phosphorylation by other kinases, such as SP-STK, may favour the progression to invasive infection^19^,^34^. However, this mechanism of transition to an invasive phenotype seems to be uncommon, since it was found in a minority of invasive isolates (10%).

The absence of expansion of isolates carrying null alleles in any of the three analysed genes and the fact that they are all under stabilising selection suggests that although mutations compromising the activity of these regulators may favour the progression to invasive disease, they are not beneficial to the overall fitness of *S. pyogenes*. Strains carrying *covRS* null alleles presented decreased ability to survive in human saliva and to persist in the murine nasopharynx, leading to the proposal that these strains would have an impaired colonisation and transmission capacity^19^,^35^. In a murine skin and soft tissue infection model, infection with a mixed population containing both the wild-type strain and a derivative *covRS* mutant resulted in higher virulence than infection with either strain alone, highlighting the importance of the presence of isolates carrying a functional CovRS^2^. Therefore, in spite of promoting a switch to a phenotype that favours the survival of GAS in deeper tissues, the loss of CovS may affect the success of the initial stages of infection^8^.

In order to evaluate the phenotypic impact of mutations in the *covRS* and *ropB* genes, we determined the activity of three extracellular proteins that are known to be involved in GAS pathogenesis and to be under the direct or indirect influence of these regulators, namely SpeB, NADase, and SLS. A functional RopB is considered an absolute requirement for *speB* transcription^13^, while the influence of *covS* mutations in *speB* expression is more controversial^2^,^5^,^7^,^8^,^17^,^18^,^19^,^32^. Our results support a role of both CovRS and RopB in the expression of SpeB, since null alleles in both *covS* and *ropB* were associated with the absence of SpeB production. Overall, the absence of SpeB activity was significantly more common among *emm* types 6 and 89, both frequently reported among invasive infections, especially *emm*89, which is one of the leading *emm* types among invasive isolates in several European countries^23^. However, the *emm* types that have been found to be significantly overrepresented among invasive infections in Portugal, namely *emm*1 and *emm*64^21^, were not associated with an increased proportion of SpeB-negative isolates. In agreement, the absence of SpeB activity was not associated with invasive infection, indicating that, in spite of preserving several virulence factors that are regarded as important for the invasive process, the abrogation of SpeB activity cannot explain by itself the higher ability of certain clones to cause invasive disease.

Several studies report a significant influence of RopB on the expression of virulence factors other than SpeB, including NADase and SLS, either directly or due to the regulation of SpeB or the interaction with other transcriptional regulators^10^,^11^,^24^. However, it has been demonstrated that the RopB regulon is highly variable, even among strains of the same *emm* type and that the core regulon is limited to the *speB* and *spi* genes^36^. Our results are in agreement with this observation, since changes in *ropB* were not significantly associated with altered NADase or SLS activities. This suggests that, despite the influence that the regulator may have in the expression of these proteins in particular lineages, in the overall GAS population and under the growth conditions used in this study, RopB does not contribute significantly to the expression of the *nga* and *sag* operons. In contrast, *covS* null alleles were significantly associated with an increased activity of both NADase and SLS, which is consistent with a repression of the corresponding operons by phosphorylated CovR, as demonstrated for the *sag* promoter^18^. However, exceptions were noted in isolates of specific *emm* types, including *emm*64, supporting the existence of differences in the CovRS regulon of distinct GAS lineages^9^. Although both NADase and SLS have been shown to contribute to GAS cytotoxicity and virulence^37^,^38^, their individual activities were not correlated with the type of infection caused by the isolates.

The association of missense alleles with invasiveness and with the activities of the studied virulence factors is harder to evaluate. Most of the alleles corresponding to amino acid changes in CovS and RopB occurred in multiple (often all) isolates of the same *emm* type, indicating that these are clonal mutations and probably do not represent the mechanism described for GAS in which mutations leading to a hypervirulent phenotype are acquired during infection^2^,^3^. Missense changes can have distinct effects on protein function depending on the nature of the amino acid replacement and the region where they occur. It is not possible to know from the current data if an altered phenotype in isolates carrying missense alleles is due to amino acid changes in these regulators or to some other characteristic of that specific lineage. Possibly reflecting this, the prevalence of missense alleles in *ropB*, *covR*, and *covS* could not be associated with the phenotypes tested. When considering only the non-clonal missense alleles, an association with SpeB-negative isolates, but not with invasive disease, was observed in the case of *ropB*. Non-clonal missense alleles in *covRS* were not associated with any particular phenotype regarding the tested virulence factors, nor with disease presentation.

Null *covS* alleles are associated with invasive isolates and present phenotypes consistent with the ablation of this regulatory system. However, none of the phenotypes is itself associated with invasiveness, indicating that other factors under *covRS* regulation may be responsible for this higher virulence. In agreement, impairment of RopB function, known to result in SpeB downregulation, is not associated with invasiveness. The data presented supports the abrogation of CovS function as a mechanism contributing to the pathogenesis of invasive GAS infections, although this is not specifically associated with lineages identified as having enhanced invasiveness, but occurred in isolates of diverse *emm* types and STs. This mechanism is uncommon and the acquisition of such changes, despite being beneficial in the specific context of invasive disease, may incur an overall fitness cost for GAS, preventing their fixation in the population.

## Materials and Methods

### Bacterial strains and culture conditions

GAS isolates used in this study (104 recovered from pharyngeal exudates of patients with pharyngitis and 87 associated with invasive disease, recovered from normally sterile sites) are listed in Supplementary Table S1 online. The strains were randomly selected among a collection of 480 non-duplicate isolates recovered from human infections between 2000 and 2005 that have been previously characterised^21^, so as to include one third of the isolates of each *emm* type present in the collection with *n* ≥ 3, as well as all isolates of *emm* types 1 and 64. Strains SF370 (CECT 5109, obtained from Colección Española de Cultivos Tipo) and MGAS5005 (BAA-947, obtained from American Type Culture Collection) were used as controls. Strains were grown at 37 °C in Todd Hewitt broth (THB) (BD, Sparks, MD, USA) or in Tryptone Soya Agar (Oxoid, Basingstoke, UK) supplemented with 5% defibrinated sheep blood.

### Molecular typing

In addition to the molecular characterisation of the isolates that had been previously performed^21^, multilocus sequence typing analysis was completed for all isolates.

### Gene sequencing and analysis

Genomic DNA of the isolates was extracted using cetyl trimethylammonium bromide (CTAB)^39^. All PCR and sequencing primers used in this study are listed in Supplementary Table S3. The sequences obtained for each isolate were assembled and compared with the corresponding regions of the genome of strain SF370 (GenBank AE004092), considered to be the reference wild-type alleles.

Geneious R7 (Biomatters, Auckland, New Zealand) was used to search the GenBank database (accessed on April 7^th^ 2014) using BLAST for all previously deposited sequences of *covR*, *covS*, and *ropB* of *S. pyogenes*, and align them with the trimmed sequences of the isolates analysed in this study, using the MUSCLE algorithm with default settings.

The subset of non-duplicate alleles without indels was used to calculate the ratio of the number of nonsynonymous substitutions per nonsynonymous site to the number of synonymous substitutions per synonymous site (*K*a/*K*s), and the respective Z test for stabilising selection, using MEGA5, with the Mega-Kumar method (Kimura 2-paramether). Values of *P* < 0.05 were considered statistically significant. Nucleotide diversity was calculated using DnaSP v.5.10.1.

### Determination of protease activity

#### Azocasein assay

SpeB protease activity in stationary phase GAS culture supernatants was determined using an adapted azocaseinolytic assay already modified for the 96-well plate format^40^,^41^. Briefly, 24 h-cultures of each strain were diluted 1:10 in fresh THB and grown for 18 h, in 96-well plates. Bacteria-free supernatants were obtained by centrifugation at 3220 × g for 10 min, and transferred to a new microtitre plate. An equal volume of activation buffer (0.1 M sodium acetate [pH 5], 1 mM EDTA, 20 mM dithiothreitol) was added and the plate was incubated for 1 h at 40 °C. After activation, 2% azocasein (Sigma-Aldrich, St. Louis, MO, USA) (w/v in activation buffer) was added and incubated for 6 h at 40 °C. Samples were then precipitated with 2.5 volumes of 6% trichloroacetic acid and centrifuged at 15000 × g for 5 min. The optical density at 450 nm of the resulting supernatants was determined. The corresponding proteolytic activities were calculated using a calibration curve performed for each plate with known concentrations of proteinase K (Roche Diagnostics). Three independent assays were performed for each strain. The presence of proteolytic activity was considered positive when at least two of the three assays showed a value ≥0.0025 U.

#### Casein-plate assay

GAS expression of extracellular cysteine protease was determined by a plate assay as previously described^16^. Briefly, single GAS colonies were stab-inoculated into plates of medium containing 0.5-strength Columbia broth (BD), 3% w/v skim milk (BD), and 1% w/v agar (Oxoid). Protease-expressing strains produced a translucent zone surrounding the site of inoculation after 24-h incubation at 37 °C. Three independent assays were performed for each strain. The presence of proteolytic activity was considered positive when at least two of the three assays presented a translucent zone of size similar to the one of strain SF370.

### SpeB Western blot analysis

For Western blot analysis, bacterial cultures were grown to late stationary phase (18 h) and centrifuged at 3200 × g for 15 min. Sterile-filtered supernatants were separated by electrophoresis on a 12% SDS-PAGE gel and transferred to a nitrocellulose membrane (Whatman, Dassel, Germany) using a Mini Trans-Blot (Bio-Rad, Hercules, CA, USA). Immunodetection was performed by chemiluminescence using monoclonal anti-SpeB antibody (Toxin Technology, Serasota, FL, USA) and goat anti-rabbit IgG conjugated with horseradish peroxidase (Santa Cruz Biotechnology, Dallas, TX, USA). Detection was performed with ECL detection reagents (Thermo Scientific, Waltham, MA, USA) according to the manufacturer’s instructions.

### Determination of NADase activity

The NADase activity of each strain was determined by an endpoint titre method based on previously published assays^37^. Briefly, bacteria-free supernatants of stationary-phase cultures of GAS strains were obtained as described for the azocasein assay and serially diluted (three-fold for the first dilution and two-fold for the second dilution onwards, up to 1/384) in β-NAD (Sigma-Aldrich) dissolved in PBS for a final concentration of 0.67 mM, in black 96-well plates (transparent bottom) (Greiner Bio-One, Frickenhausen, Germany). Wells containing sterile THB serially diluted in PBS and in β-NAD/PBS were used as controls, for each plate. After incubation for 1 h at 37 °C in the dark, an equal volume of NaOH was added, for a final concentration of 2 N, and the plates were incubated for 1 h at room temperature, in the dark. Fluorescence was measured with excitation at 340 nm and emission detection at 460 nm. For each strain, the NADase activity was expressed as the inverse of the highest dilution prior to a greater or equal to two-fold increase in the fluorescence value. This method has a limit of detection of 3, and strains for which a two-fold increase was not observed and with values similar to the THB + β-NAD control were considered to have a NADase activity of ≤3. A minimum of three independent assays were performed for each strain, and the majority rule was used to determine the final NADase activity.

### Determination of streptolysin (SLS) activity

The streptolysin activity of each strain was determined by an endpoint titre method adapted from a previously published assay^20^. Briefly, bacteria-free supernatants of stationary-phase cultures of GAS strains were obtained as described for the azocasein assay. The supernatants (or sterile THB for control of spontaneous haemolysis – blank) were serially diluted in PBS in microplate wells (three-fold for the first dilution and two-fold for the second dilution onwards, up to 1/192), and an equal volume of a 2.5% (v/v) suspension of defibrinated sheep erythrocytes was added. Two complete haemolysis controls were included in each plate, by incubating the erythrocytes suspension with 1% Triton X-100 (positive controls). The blank for these wells consisted only of PBS and the erythrocyte suspension. After incubation at 37 °C for 1 h, erythrocytes were pelleted and the absorbance at 570 nm of the supernatants was measured. For each well, the percentage of haemolysis relative to the positive control was calculated as follows:


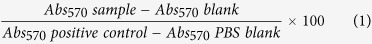


The streptolysin activity was expressed as the inverse of the highest dilution prior to a ≥ two-fold decrease in the percentage of haemolysis. This method has a limit of detection of 3, and strains for which a two-fold decrease was not observed were considered to have a streptolysin activity of ≤ 3. A minimum of three independent assays were performed for each strain, and the majority rule was used to determine the final streptolysin activity. In four strains, two with functional *covRS* alleles (SF370 and SH0959A in Supplementary Table S1) and another two, of the same *emm* types, with null *covS* alleles and increased NADase activity (SH1025A and SH0421A in Supplementary Table S1), streptolysin assays were also performed in the presence of 33.3 μg/ml of trypan blue (Sigma-Aldrich) or of 16.7 μg/ml cholesterol (Sigma-Aldrich). In all four strains, the results were not significantly changed by the presence of cholesterol, while trypan blue completely inhibited haemolysis, indicating that streptolysin O does not contribute to the haemolytic activity determined under these conditions, in agreement with previous reports^20^.

### Statistical analysis

The allelic diversity of the *covR*, *covS*, and *ropB* genes in the studied collection of GAS isolates was evaluated using the Simpson’s index of diversity (SID) and corresponding 95% confidence intervals (CI_95%_)^42^. The overall association between *covR*, *covS*, or *ropB* alleles and the *emm* type and ST was evaluated with the Adjusted Wallace coefficient with corresponding CI_95%_^43^.

Unless otherwise specified, the statistical significance of pairwise associations was evaluated by calculating the respective odds ratios (when applicable) and the two-tailed Fisher’s exact test, correcting the *P* values for multiple testing through the FDR linear procedure^44^.

## Additional Information

**How to cite this article**: Friães, A. *et al.* Consequences of the variability of the CovRS and RopB regulators among *Streptococcus pyogenes* causing human infections. *Sci. Rep.* **5**, 12057; doi: 10.1038/srep12057 (2015).

**Accession codes**: All new covRS and ropB sequences identified in this study were deposited in GenBank (accession numbers KM985476 to KM985497 and KP101294 to KP101323).

## Supplementary Material

Supplementary Information

## Figures and Tables

**Table 1 t1:** Type of amino acid alteration, nucleotide diversity, and *Ka*/*Ks* values associated with the *covR, covS*, and *ropB* alleles described in this study and those previously reported in GenBank.

Gene	No. alleles (this study)	No. amino acid sequences (this study)	No. alleles with indels	No. alleles with nonsense mutations	Nucleotide diversity	*K*a/*K*s	*P* value
*covR*	54 (19)	32 (5)	1	2	0.0058	0.19	0.0032
*covS*	121 (43)	83 (26)	28^a^	11	0.0043	0.14	0.0029
*ropB*	78 (37^c^)	56 (21^c^)	7^b^	2	0.0055	0.23	0.0015

^a^Three of the *covS* indels are in frame.

^b^Two of the *ropB* indels are in frame, but one of these deletes a considerable portion of the protein (119 residues).

^c^Excluding the isolate with a complete *ropB* deletion.

**Table 2:** Simpson’s index of diversity (SID) and corresponding 95% confidence intervals (CI _95%_) of the alleles of *covR, covS*, and *ropB*, the *emm* types, and the STs identified among the 191 GAS isolates analysed in this study.

Gene	All isolates (*n* = 191)		Invasive isolates (*n* = 87)		Pharyngitis isolates (*n* = 104)	
	No. partitions	SID [CI_95%_]	No. partitions	SID [CI_95%_]	No. partitions	SID [CI_95%_]
*covR*	19	0.831 [0.793–0.870]	17	0.816 [0.752–0.880]	13	0.839 [0.794–0.885]
*covS*	43	0.893 [0.861–0.925]	31	0.870 [0.811–0.930]	25	0.905 [0.873–0.938]
*ropB*	38	0.850 [0.805–0.896]	23	0.806 [0.729–0.883]	29	0.879 [0.828–0.929]
*emm* type	26	0.877 [0.844–0.910]	20	0.833 [0.769–0.898]	20	0.900 [0.868–0.932]
ST	41	0.894 [0.861–0.927]	27	0.860 [0.798–0.922]	32	0.910 [0.876–0.945]

**Table 3:** Number of isolates from invasive infections and pharyngitis presenting null and missense alleles in the *covR*, *covS*, and *ropB* genes.

	Allele type	Invasive (*n* = 87)	Pharyngitis (*n* = 104)
*covR*	Null	0	0
	Missense	2	2
*covS*	Null	9	2
	Missense	51	61
*ropB*	Null	3	3
	Missense	13	26
*covRS/ropB*	Null	12	5
	Missense	56	72
