# Supplementary material for: Consequences of the variability of the CovRS and RopB regulators among Streptococcus pyogenes causing human infections
Source: Sci Rep. 2015 Jul 15;5:12057. doi: 10.1038/srep12057 (PMC4502508; doi:10.1038/srep12057)
Supplement: Supplementary Information [file srep12057-s1.pdf]

Consequences of the variability of the CovRS and RopB regulators among *Streptococcus pyogenes* causing human infections

Ana Friães[†], Catarina Pato[†], José Melo-Cristino, Mario Ramirez[*]

Instituto de Microbiologia, Instituto de Medicina Molecular, Faculdade de Medicina, Universidade de Lisboa, Lisbon, Portugal

[†]A.F. and C.P. have contributed equally to this work.

[*]Corresponding author:

Mario Ramirez

Instituto de Microbiologia
Faculdade de Medicina,
Universidade de Lisboa
Av. Prof. Egas Moniz
1649-028 Lisboa
PORTUGAL

ramirez@fm.ul.pt

Table S1 – Isolation source, molecular typing data, results of *covRS* and *ropB* sequencing, and results of SpeB, NADase, and SLS activity obtained for the 191 GAS isolates studied.

http://dx.doi.org/10.6084/m9.figshare.1328335

Table S2 – Adjusted Wallace values (95% confidence intervals) of the *emm* type and the ST towards the *covR*, *covS*, and *ropB* alleles among the 191 GAS isolates analyzed in this study.

	covR	*covS*	*ropB*
emm type	0.978 (0.962-0.994)	0.834 (0.741-0.928)	0.806 (0.723-0.888)
ST	0.988 (0.974-1.000)	0.854 (0.753-0.955)	0.824 (0.735-0.913)

Table S3 – Primers used for the amplification and sequencing of the *covRS* and *ropB* genes.

Primer Name	Sequence (5’→3’)
covRS-1	TAACCTCGAAGAAAGTATTGTGG
covRS-4	CAACACGCTCAAAGGTAGTAAAG
covRS-5	GTTTGCCAGTCACTGAAAGG
covRS-6	GGATTTTCAGAGATATTAC
covRS-7	AATCAGTGTAAAGGCAGAG
ropB-F	GATAAAACTATCGCATCTGGC
ropB-R	CCTGGAGCTGTTGAGATAAAC
